# A mammosphere formation RNAi screen reveals that ATG4A promotes a breast cancer stem-like phenotype

**DOI:** 10.1186/bcr3576

**Published:** 2013-11-14

**Authors:** Jonas Wolf, Dyah Laksmi Dewi, Johannes Fredebohm, Karin Müller-Decker, Christa Flechtenmacher, Jörg D Hoheisel, Michael Boettcher

**Affiliations:** 1 Division of Functional Genome Analysis, Deutsches Krebsforschungszentrum, Heidelberg, Germany; 2 Core Facility Tumor Models, Deutsches Krebsforschungszentrum, Heidelberg, Germany; 3 Institute of Pathology, University Hospital, Heidelberg, Germany

## Abstract

**Introduction:**

Breast cancer stem cells are suspected to be responsible for tumour recurrence, metastasis formation as well as chemoresistance. Consequently, great efforts have been made to understand the molecular mechanisms underlying cancer stem cell maintenance. In order to study these rare cells *in-vitro*, they are typically enriched via mammosphere culture. Here we developed a mammosphere-based negative selection shRNAi screening system suitable to analyse the involvement of thousands of genes in the survival of cells with cancer stem cell properties.

**Methods:**

We describe a sub-population expressing the stem-like marker CD44^+^/CD24^-/low^ in SUM149 that were enriched in mammospheres. To identify genes functionally involved in the maintenance of the sub-population with cancer stem cell properties, we targeted over 5000 genes by RNAi and tested their ability to grow as mammospheres. The identified candidate ATG4*A* was validated in mammosphere and soft agar colony formation assays. Further, we evaluated the influence of ATG4*A* expression on the sub-population expressing the stem-like marker CD44^+^/CD24^low^. Next, the tumorigenic potential of SUM149 after up- or down-regulation of ATG4*A* was examined by xenograft experiments.

**Results:**

Using this method, Jak-STAT as well as cytokine signalling were identified to be involved in mammosphere formation. Furthermore, the autophagy regulator ATG4A was found to be essential for the maintenance of a sub-population with cancer stem cell properties and to regulate breast cancer cell tumourigenicity *in vivo.*

**Conclusion:**

In summary, we present a high-throughput screening system to identify genes involved in cancer stem cell maintenance and demonstrate its utility by means of ATG4*A*.

## Introduction

Breast tumours, like many other solid tumours, consist of highly heterogeneous cell populations with varying phenotypic and functional properties [1]. Similar to the normal mammary gland, these populations include cells with luminal-epithelial, basal and stem-cell-like features [2]. Based on gene expression profiles, basal-like breast cancers have been associated with the surface marker expression CD44^+^/CD24^-/low^[3] while luminal epithelial cells have been associated with CD24^+^/CD44^-^ expression [4,5]. Stem-like cells with tumour initiating capabilities have been found to be enriched in the CD44^+^/CD24^-/low^ sub-population of basal breast carcinoma cells [6]. These stem-like cells or cancer stem cells (CSCs) are held responsible for metastasis formation [7,8] and chemoresistance [9]. Further, it was found that CD44^+^/CD24^-/low^ breast cancer cells exhibit epithelial-to-mesenchymal transition (EMT) features that might be responsible for their aggressive clinical behaviour [10]. EMT has long been recognised as an important programme for embryonic development [11] and has more recently been associated with breast CSC regulation [12,13]. It is hypothesised that differentiated cancer cells can become CSCs as a consequence of EMT, allowing them to migrate, metastasize and survive chemotherapy [7,14]. In line with these findings, CSCs have been connected to a mesenchymal phenotype [15], and it was shown that chemoresistant cells display not only CSC but also mesenchymal features [14]. Due to the aggressive nature of CSCs, the identification of genes and pathways that they depend on is an active area of research, fuelled by the promise that combination of conventional chemotherapy with specific CSC inhibitors will increase therapeutic success rates [16].

A major restraint when studying stem cells as well as CSC is their rareness. One approach to enrich breast stem cells, which has become particularly popular over the past ten years, is culturing cells as mammospheres [17]. It was shown that rare, single-founder stem cells can form multi-cellular sphere structures under serum-free suspension conditions that are enriched for stem and early progenitor cells [17]. Later, it was also found that rare cancer cells possess the ability to form mammospheres enriched for highly tumourigenic CSCs of the CD44^+^/CD24^-/low^ phenotype [5,18]. Moreover it was shown that cells enriched in mammospheres had passed through EMT and were chemoresistant [19] which are two properties typically attributed to CSCs [12]. Here, we utilised the enrichment of CSCs in mammospheres and developed a high-throughput a short hairpin RNA interference (shRNAi) screening system to assay the involvement of over 5,000 genes in the maintenance of a population of cells with CSC properties. The results give an insight into molecular mechanisms underlying CSC maintenance in mammospheres and attribute a previously unrecognised role in this process to the autophagy regulator ATG4A.

## Materials and methods

### Adherent and mammosphere cell culture

SUM149 cells were obtained from the Kuperwasser Laboratory (Tufts University, Boston) and are commercially available (Asterand, Royston, UK). Cells were cultured in Ham’s F12 (Life Technologies, Darmstadt, Germany) with 5% calf serum, 5 μg/ml bovine insulin (Sigma-Aldrich, Taufkirchen, Germany), and 1 μg/ml hydrocortisone (Sigma-Aldrich). MDA-MB-231 and MCF-7 were cultured in DMEM (Life Technologies) with 10% calf serum. For mammosphere formation, 10^4^ cells/cm^2^ cells were plated in an ultra-low attachment cell culture flask (Corning, Kaiserslautern, Germany) and cultured in MammoCult medium (StemCell Technologies, Grenoble, France). After 14 days, the mammospheres were counted and pictures were taken. For flow cytometric analysis, spheres were filtered through a 40-μm cell strainer (Becton Dickinson, Heidelberg, Germany) and treated with Accutase (Sigma-Aldrich) to obtain a single cell suspension.

### Mammosphere formation RNAi screen

The DECIPHER RNAi library Module 1 (Cellecta, Mountain View, USA) [20] was used at low multiplicity of infection (MOI = 0.3) to transduce SUM-149 cells, followed by 48 h of selection with 2.5 μg/ml puromycin. Following 48 h recovery in antibiotic-free medium, 1.4 × 10^8^ stably transduced cells were seeded into 180 × 75 cm^2^ ultra-low attachment cell culture flask (Corning) and cultured in MammoCult (StemCell Technologies). After 14 days, mammospheres larger than 40 μm were extracted by five individual rounds of filtration through 40-μm mesh size cell strainers (Becton Dickinson). About 7 × 10^6^ transduced cells were cultured adherently for the same period of time in Ham’s F12 as a reference. Cells were passaged after reaching 80% confluence. Genomic DNA from adherently cultured cells at the beginning (baseline) and the end of the screen (t_adherent_) as well as from pooled mammosphere samples (t_sphere_) was isolated using the DNeasy Blood and Tissue Kit (Qiagen, Hilden, Germany). Construct-specific barcode sequences were amplified under PCR conditions provided by the manufacturer of the DECIPHER library (Cellecta). Barcode sequences are 18 nucleotide-long DNA sequences that are unique for each of the 27,500 shRNA expression constructs in the DECIPHER library pool. Hence, they can be used as surrogate markers to quantify the number of cells expressing a certain shRNA in a pool of cells. PCR amplification of barcode sequences resulted in ready-to-load sequencing libraries including adaptor sequences for Illumina GA and HiSeq platforms. The barcodes were amplified and sequenced in duplicate on Illumina GAIIx machines and quantified using Barcode Deconvoluter software (Cellecta).

### Data analysis

Two separate barcode read-count ratios were calculated. In order to identify shRNAs, which are toxic to adherent cells or mammospheres, the ratios [t_adherent_/baseline] or [t_sphere_/baseline] were calculated, respectively. Results are shown in Additional file 1. Ratios from each set of shRNAs (5 to 6) targeting a particular gene were compared to ratios from a set of 21 negative control shRNAs targeting the gene *Luciferase* via unpaired, two-sided, unequal variance *t*-test statistics. Calculated mean fold changes from each set of shRNA expression constructs and corresponding *P*-values for every gene present in the library are shown in Additional file 2.

### Lentivirus-mediated RNAi

For target validation, shRNA template sequences identified in the screen were synthesized individually (Sigma-Aldrich) and cloned into the pRSI9 vector backbone. Cloning and virus production were performed following the protocol provided by the manufacturer (Cellecta). Sequences were as follows: shATG4A-1 5′-ACCGGCAGATACAGATGAGTTGGTATGTTAATATTCATAGCATACCAGCTCATCTGTATCTGTTTT-3′; shATG4A-2 5′-ACCGGCCCGGAAAGAAATAGAATAATGTTAATATTCATAGCATTGTTCTATTTCTTTCCGGGTTTT-3′; shATG4A-3 5′- ACCGGGCTGTTGTAATGAGGAAATGGGTTAATATTCATAGCCCATTTCCTCATTGCAGCAGCTTTT-3′ and shCTRL 5′-ACCGGATCACAGAATCGTCGTATGTAGTTAATATTCATAGCTGCATACGACGATTCTGTGATTTTT-3′. For virus production, cloned shRNA plasmids were co-transfected with the packaging plasmids psPAX2 and pMD2.G into HEK293T cells. Viral supernatant was harvested 48 h post transfection and cleared from debris via centrifugation. Cells were transduced with lentivirus for 24 h in cell culture medium containing 8 μg/ml polybrene (Sigma-Aldrich) and selected with 2.5 μg/ml puromycin (Sigma-Aldrich) for 48 h. Following selection cells were recovered for 48 h in antibiotic-free culture medium.

### mRNA quantification

Total RNA was isolated from cells or mammospheres using RNeasy Mini Kit (Qiagen). Reverse transcription and PCR were performed using the One Step Quantifast SYBR Green RT-PCR Kit (Qiagen) with a LightCycler 480 system (Roche, Mannheim, Germany). For target gene amplification, the QuantiTect Primer Assay (Qiagen) was used. Target gene expression was normalised to the expression of glyceraldehyde-3-phosphate dehydrogenase (GAPDH).

### Protein quantification

To determine the protein concentration, cells were lysed in TBS containing 1% Triton X-100, 10 mM Na_3_VO_4_, 1 mM NaF, 4 mM ethylenediaminetetraacetic acid (EDTA), protease inhibitor mixture (Roche). the protein concentration was measured with the bicinchoninic acid (BCA) Protein Assay (Thermo Fisher Scientific, Bonn, Germany). Proteins were separated by SDS-PAGE and blotted onto a nitrocellulose membrane (GE Healthcare, Freiburg, Germany). The membrane was probed with antibodies; peroxidase-conjugated secondary antibodies detected the bands by ECL Plus (ThermoFisher Scientific). Antibodies were: anti-ATG4A (GeneTex, Irvine, USA) and anti-Tubulin (Abcam, Cambridge, UK).

### Flow cytometry

vSingle cell suspension of adherent cells or spheres was stained with CD24-FITC, CD44-PE/Cy7 and EpCAM-APC (Becton Dickinson), E-cadherin-PE (Miltenyi Biotec, Bergisch Gladbach, Germany) and Vimentin-Alex488 (Cell Signaling, Danvers, USA). The cells were measured using a FACS-Canto-II (Becton Dickinson) and data were analysed using FlowJo software (Tree Star, Ashland, USA).

### Colony formation assay

We suspended 2,500 cells/cm^2^ in 0.3% agarose with MammoCult medium (StemCell Technologies) on a 0.8% agar base layer. The culture was covered with 0.5 ml MammoCult medium and cultured for 14 days. For quantification, the wells were imaged using a microscope, and the colonies were analysed using ImageJ software.

### Microarray and gene expression analysis

SUM149 cells were cultured adherently and under mammosphere formation conditions in biological triplicates for two weeks. Spheres were filtered using a 40-μm cell strainer (Becton Dickinson) and RNA was isolated from spheres and adherent cells using RNeasy Mini Kit (Qiagen). RNA was analysed on HumanHT-12 v4 Expression BeadChip (Illumina, San Diego, USA) according to manufacturer instructions. Raw data were normalised and grouped using Chipster. Genes with significant gene expression changes (*P* <10^-10^) were used for pathway enrichment analysis using DAVID Functional Annotation Tool [21]. Data were uploaded to ArrayExpress [22] under the accession number E-MTAB-1553.

### MACS cell enrichment of sub-population

The described sub-population of SUM149 cell was enriched by depletion of EpCAM-expressing cells using EpCAM MicroBead Kit (Miltenyi Biotec). The depletion was performed according to the manufacturer’s protocol. Enrichment of CD44^+^/CD24^low^/EpCAM^-/low^ cells was confirmed via fluorescent-activated cell sorting (FACS).

### Xenograft experiments

Cells were transduced with plasmids expressing shATG4A-1 and -2 (shATG4A), the ATG4A open reading frame (ATG4A-OE), or a non-silencing control (shCTRL). This was followed by a selection of transduced cells with puromycin. For each injection, 4 × 10^4^ cells in 15 μl PBS were mixed 1:1 (v/v) with Matrigel (BD Biosciences, Heidelberg, Germany) prior to injection into the second left thoracic mammary fat pad of 8- to 9-week-old NOD SCID gamma (NSG) female mice. Tumour growth was monitored over a period of 15 weeks and tumour size was determined twice a week using a caliper. Significance values from Kaplan-Meier plots were calculated using the Wilcoxon test and GraphPad Prism software. For tissue staining, tumours were collected and embedded into paraffin according to routine procedures. H&E staining was done on 5-μm paraffin sections. Studies were approved by the local ethics committee at Regierungspräsidium Karlsruhe (G74/11, G244/11).

## Results

### SUM-149 cell line contains a sub-population of cells with cancer stem-cell properties

**Figure 1:**
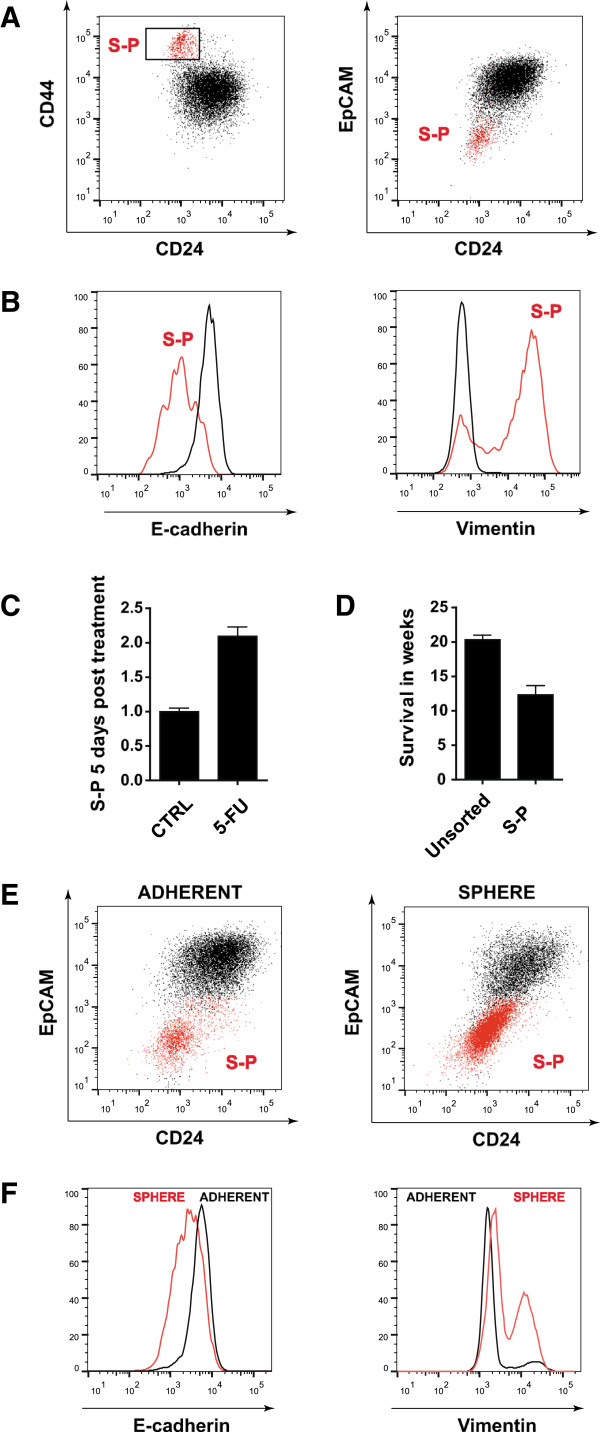
**The SUM-149 cell line contains a sub-population of cells with cancer stem-cell (CSC) properties. (A)** Gated and highlighted in red are cells expressing the stem-like marker signature CD44^+^/CD24^low^. CD44^+^/CD24^low^ cells also express low levels of EpCAM. Cells were cultured under adherent conditions. A sub-population (S-P) of cells with CSC properties is shown in red; a luminal epithelial population is shown in black. **(B)** Expression of epithelial marker E-cadherin and mesenchymal marker vimentin in S-P (red) compared to the luminal population (black). **(C)** Enrichment of S-P five days post treatment with 5′-fluorouracil (5′-FU) relative to untreated cells (CTRL). **(D)** Survival of NOD SCID gamma (NSG) mice following subcutaneous injection of 6,000 cells from S-P or unsorted SUM-149 cells. **(E)** Enrichment of stem-like CD44^+^/CD24^low^/EpCAM^-/low^ cells (highlighted in red) in mammospheres. **(F)** Expression of E-cadherin and vimentin in adherently cultured cells compared to mammospheres.

Flow cytometry analysis of the triple-negative human breast cancer cell line SUM-149 revealed two distinct sub-populations of cells. As previously described [5], we confirmed the existence of a small sub-population (S-P) of cells expressing the stem-like marker signature CD44^+^/CD24^low^ (Figure 1A). It was found that CD44^+^/CD24^low^ cells also express low levels of the epithelial cell adhesion molecule EpCAM (Figure 1A). This CD44^+^/CD24^low^/EpCAM^-/low^ population was previously demonstrated to have basal as well as stem-like features, while the opposing CD44^-^/CD24^+^/EpCAM^+^ population was described to be luminal [23]. To further examine both populations for epithelial or mesenchymal phenotypes, the expression of two markers commonly used to detect EMT, namely E-cadherin [24] and vimentin [25], was analysed in both populations. It was shown that cells from the sub-population were almost completely negative for the epithelial marker E-cadherin and expressed higher levels of the mesenchymal marker vimentin (Figure 1B) when compared to the luminal population. Moreover, five days treatment of SUM-149 cells with the chemotherapeutic drug 5′-fluorouracil (5′-FU) resulted in an enrichment of cells from the sub-population (Figure 1C). Last, sorted cells from the sub-population injected subcutaneously into NSG mice formed tumours much more rapidly than unsorted SUM-149 cells (Figure 1D). Taken together, the characterised sub-population of cells displays several CSC properties, namely expression of stem-like surface markers, passage through EMT, and chemoresistance, as well as increased tumourigenicity *in-vivo*[12].

## Cells with cancer stem-cell properties accumulate in mammospheres

It was previously shown that cancer cells with stem-like characteristics become strongly enriched in mammospheres [17]. This enrichment is a result of their ability to grow independently of anchorage, a condition under which most cancer cells undergo anoikis [26]. The resistance to anoikis is commonly attributed to cells that have undergone EMT [13,26]. As shown in Figure 1E, flow cytometry analysis of mammosphere-derived SUM-149 cells revealed an enrichment of the CD44^+^/CD24^low^/EpCAM^-/low^ population compared to adherent cultured cells. In accord with the enrichment of this sub-population in mammospheres, it was found that spheres express lower levels of E-cadherin and higher levels of vimentin when compared to adherent cells (Figure 1F). These data clearly confirmed that a sub-population of cells with CSC properties became enriched during mammosphere formation. Therefore, targeting the survival of these cells should lead to impaired sphere formation. Based on this hypothesis, we established a screening system for the identification of genes that are specifically involved in mammosphere formation.

## Negative selection shRNAi screen for specific regulators of mammosphere formation

**Figure 2:**
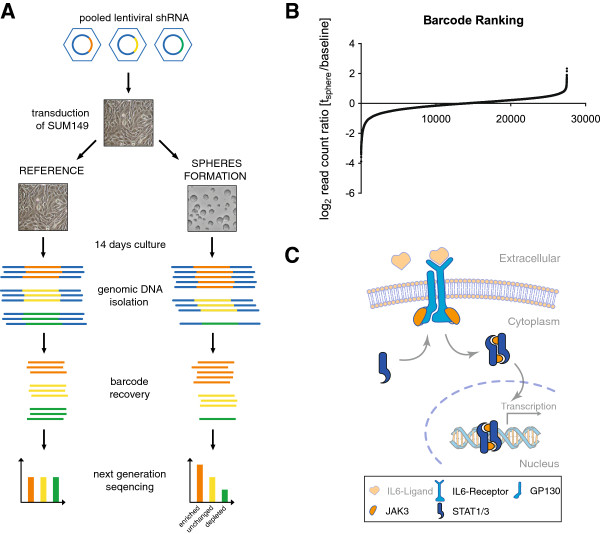
**Negative selection mammosphere formation shRNAi screen. (A)** Schematic, illustrating layout of pooled RNAi screen. Cells were transduced with the lentiviral DECIPHER RNAi library pool at low multiplicity of infection and cultured for fourteen days under adherent or suspension conditions, respectively. At the end of the screen, adherent cells and mammospheres >40 μm were harvested. Using next-generation sequencing of barcodes, the abundance of each shRNA expression construct in the pool was determined at the beginning of the screen (baseline) and from adherently cultured cells and mammospheres at fourteen days post seeding. Colour scheme: red: toxic shRNA; yellow: shRNA differentially inhibiting sphere formation; green: shRNA differentially activating sphere formation. **(B)** Barcode ranking. Shown are log_2_ (t_sphere_/baseline) read-count ratios for all 27,500 shRNA expression constructs analysed in the screen. **(C)** Selected candidate genes involved in Janus kinase (Jak)-signal transducers and activators of transcription (STAT) Jak-STAT signalling.

**Figure 3:**
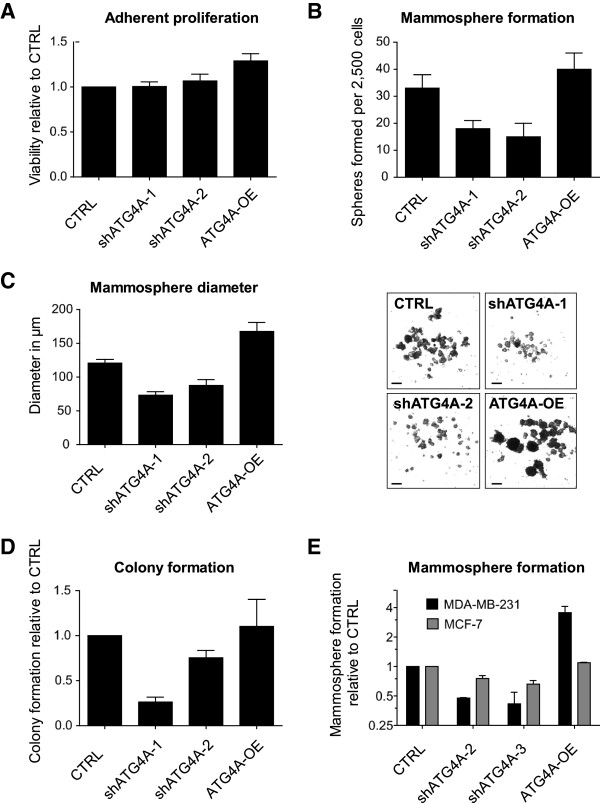
**ATG4A expression regulates mammosphere formation in breast cancer cell lines. (A)** Viability of SUM-149 cells cultured under adherent conditions with ATG4A inhibition or overexpression. **(B)** Number of mammospheres formed fourteen days post seeding 2,500 SUM-149 cells in serum-free suspension conditions. **(C)** Average size of SUM-149 mammospheres fourteen days post seeding and representative images of sphere numbers and sizes. Scale bars are 200 μm. **(D)** Relative number of colonies in soft agar fourteen days post seeding of SUM-149 cells. **(E)** Relative number of mammospheres at fourteen days post seeding of basal MDA-MB-231 or luminal MCF-7 cells with inhibited (shATG4A) or increased ATG4A expression (ATG4A-OE). CTRL, control.

SUM-149 cells were transduced with the pooled, lentiviral DECIPHER library Module 1 under conditions that ensured a maximum of one integration event per cell. The module targets 5,045 genes for knockdown by 5 to 6 dissimilar shRNA sequences per target gene, adding up to a total of 27,500 shRNA expression constructs that integrated into the genome of the host cells. In order to identify genes whose inhibition selectively impairs the formation of mammospheres, cells were sub-cultured under two distinct conditions. One fraction of cells was cultured adherently and a second fraction was cultured under mammosphere formation conditions (Figure 2A). After fourteen days in suspension culture, 1.3 percent of cells formed mammospheres (Figure 3B) with an average size of 120 μm (Figure 3C). A total of 1.8 million mammospheres or 66 spheres per shRNA were analysed in the screen. To identify shRNAs with expression that impaired sphere formation, mammospheres larger than 40 μm were collected, and smaller spheres and single cells were discarded (Figure 2A). From cells at the beginning of the screen (baseline), cells cultured adherently for fourteen days (t_adherent_) and mammospheres larger 40 μm (t_sphere_), barcode sequences were recovered using PCR and quantified using next-generation sequencing. Each barcode sequence stands for a particular shRNA expression construct. Figure 2B shows barcode read-count ratio distributions from sphere-cultured cells relative to the baseline. Corresponding values are given in Additional file 1. Based on those values, the impact of every single gene on adherent proliferation as well as mammosphere formation was determined and the results are shown in Additional file 2. In a first analysis step, a set of 1,015 genes was identified the inhibition of which significantly impaired the adherent survival of cells (*P* <0.01). Pathway enrichment analysis using the DAVID Functional Annotation Tool [21] revealed highest enrichment of identified genes in *Kyoto Encyclopedia of Genes and Genomes* (KEGG) pathways related to proteasomal and ribosomal function (Table 1A). Although inhibition of the majority of those genes also impaired mammosphere formation, they cannot be considered to inhibit this process selectively. Consequently, in a second analysis step, only genes that impaired mammosphere formation (*P* <0.01) but had no impact on adherent proliferation (*P* >0.1) were used for pathway enrichment analysis. Pathway analysis showed the highest enrichment of candidate genes in Janus kinase (Jak)-signal transducers and activators of transcription (STAT) and cytokine signalling followed by mTOR and several cancer-related signalling pathways (Table 1B). Genes associated with each pathway are shown in Additional file 3. As an example, five identified key regulators acting in Jak-STAT signalling are summarised in the scheme shown in Figure 2C.

**Table 1:** Pathway enrichment analysis of candidate genes

**KEGG pathway**	**Fold enrichment**	***P-value***
**A**		
hsa03050:Proteasome	3.38	*1.41E-08*
hsa03010:Ribosome	3.89	*2.28E-06*
hsa04114:Oocyte meiosis	1.77	*1.64E-03*
hsa04914:Progesterone-mediated oocyte maturation	1.71	*5.03E-03*
hsa03040:Spliceosome	2.39	*8.61E-03*
hsa05110:Vibrio cholerae infection	1.86	*1.55E-02*
hsa04110:Cell cycle	1.49	*1.76E-02*
hsa05016:Huntington’s disease	1.49	*1.76E-02*
hsa03020:RNA polymerase	2.27	*1.96E-02*
hsa00970:Aminoacyl-tRNA biosynthesis	1.97	*2.08E-02*
hsa00730:Thiamine metabolism	3.57	*3.30E-02*
hsa04260:Cardiac muscle contraction	1.96	*3.80E-02*
hsa00240:Pyrimidine metabolism	1.52	*4.51E-02*
hsa00020:Citrate cycle (TCA cycle)	1.90	*4.80E-02*
**B**		
hsa04630:Jak-STAT signaling pathway	2.05	*1.76E-03*
hsa04060:Cytokine-cytokine receptor interaction	1.67	*7.08E-03*
hsa05215:Prostate cancer	2.22	*8.50E-03*
hsa04150:mTOR signaling pathway	2.72	*1.47E-02*
hsa05223:Non-small cell lung cancer	2.49	*1.61E-02*
hsa05200:Pathways in cancer	1.48	*1.96E-02*
hsa04510:Focal adhesion	1.70	*2.22E-02*
hsa04062:Chemokine signaling pathway	1.63	*3.91E-02*
hsa05216:Thyroid cancer	3.04	*4.16E-02*
hsa04640:Hematopoietic cell lineage	1.88	*4.97E-02*

**A,***Kyoto Encyclopedia of Genes and Genomes* (KEGG) pathway enrichment analysis was performed for genes essential for the survival of SUM-149 cells under adherent conditions; **B,** KEGG pathway enrichment analysis was done for genes specifically involved in mammosphere formation but not adherent cell survival. Jak-STAT, Janus kinase-signal transducers and activators of transcription.

### Mammospheres express high levels of lysosomal and oxidative phosphorylation genes

In order to further investigate molecular differences between mammospheres and adherently cultured cells, gene expression profiles were compared; the results of a pathway enrichment analysis are summarized in Additional file 4. Genes involved in cell cycle regulation (P = 2 × 10^-20^) as well as DNA replication (P = 2 × 10^-14^) were found to be down-regulated in mammospheres, which is in accordance with the reduced growth rate that cells exhibit under serum-free suspension conditions. Interestingly, strongest enrichment of up-regulated genes was seen for lysosome related genes (P = 2 × 10^-18^) and genes involved in oxidative phosphorylation (P = 3 × 10^-14^) indicating a requirement of lysosomal activity and energy generation under sphere forming conditions.

### ATG4A is upregulated in mammospheres

**Figure 4:**
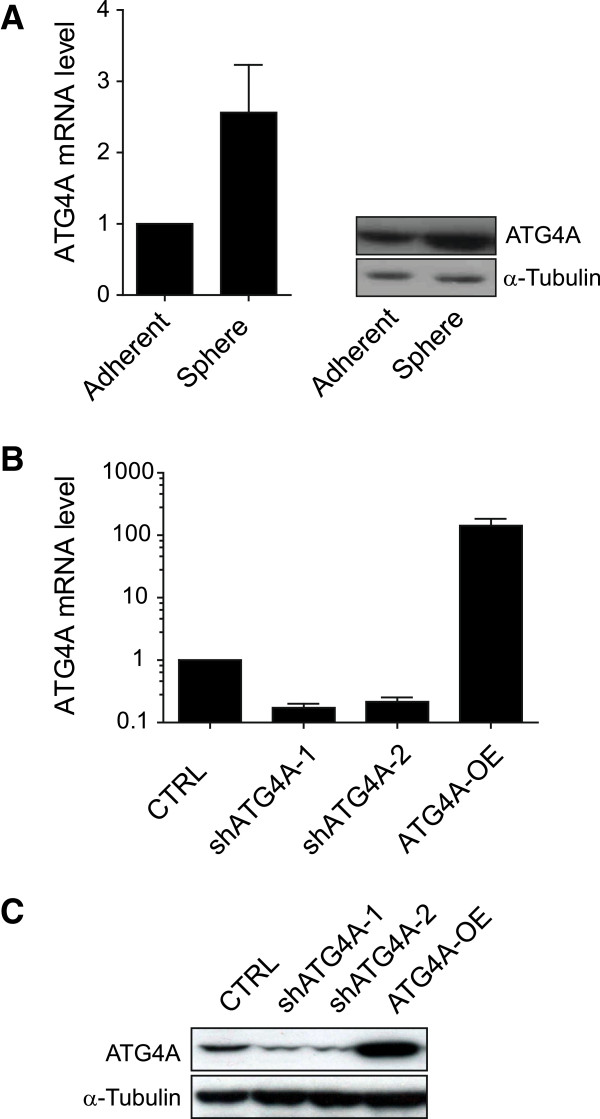
**ATG4A expression levels in SUM-149 cells. (A)** ATG4A mRNA and protein level in cells cultured adherently or as mammospheres. **(B)** Residual mRNA level of ATG4A following inhibition (shATG4A-1/-2) or overexpression (ATG4A-OE). **(C)** Residual protein level of ATG4A following inhibition or overexpression. CRTL, control.

Two candidate genes identified by the screen to be necessary for mammosphere formation were the regulators of autophagy, ATG4*A* and ATG4*B*. It was further found that mRNA and protein levels of ATG4*A*, but not ATG4*B*, were elevated in spheres when compared to adherently cultured cells (Figure 4A). Together these findings implicated an important function for ATG4*A* during mammosphere formation. Hence, this gene was selected for further investigations. In order to analyse the impact of ATG4*A* on sphere formation and maintenance of cells with a CSC phenotype, two different shRNA sequences identified by the screen as well as the open reading frame of ATG4*A* were cloned into expression vectors. As shown in Figure 4B, the expression of both shRNAs reduced ATG4*A* mRNA levels by 4-fold, whereas ATG4*A* overexpression increased mRNA levels to over 100-fold. Concurrently, ATG4*A* protein levels were found to be reduced following expression of both shRNAs and increased for ATG4*A* overexpression (Figure 4C). Hence, these expression constructs represent efficient tools to artificially modulate ATG4A expression.

### Modulation of ATG4*A* specifically regulates mammosphere formation

To investigate whether regulation of ATG4*A* specifically regulates mammosphere formation, the impact of ATG4*A* modulation on the adherent proliferation, sphere formation and sphere diameter of SUM-149 cells was determined (Figure 3A-C). It was found that inhibition of ATG4*A* had no impact on cell viability under adherent culture conditions illustrating that ATG4*A* is not an essential gene for the bulk of SUM-149 cells (Figure 3A). Yet the inhibition of ATG4*A* led to a decrease in sphere number and size. On average, 33 mammospheres formed from 2,500 cells (1.3%) seeded under serum-free suspension conditions. Inhibition of ATG4*A* reduced this figure to 18 and 15 spheres, respectively, and overexpression increased the number of spheres formed to 40 (Figure 3B). Mammospheres had an average diameter of 120 μm at fourteen days post seeding of control cells. Inhibition of ATG4*A* reduced sphere size to 73 μm or 88 μm, respectively, and ATG4*A* overexpression resulted in significantly larger spheres of 168 μm (Figure 3C). Representative images of mammospheres following ATG4*A* knockdown or overexpression for fourteen days are shown in Figure 3C. In addition to sphere formation, the colony formation capacity of SUM-149 cells seeded in soft agar was determined after up- or down-regulation of ATG4*A*. As shown in Figure 3D, inhibition of ATG4*A* reduced the number of colonies formed, and overexpression slightly increased it. Further, the impact of ATG4*A* expression on sphere formation of breast cancer cell lines from different sub-types, namely basal MDA-MB-231 (CD44^+^/CD24^-^) and luminal MCF-7 cells (CD44^-^/CD24^+^) [5] was analysed. MDA-MB-231 is a highly metastatic cell line with a high tumourigenicity compared to the non-invasive MCF-7 [27]. It was found that ATG4*A* inhibition reduced sphere formation in MDA-MB-231 cells, whereas its overexpression led to a dramatic increase (Figure 3E). Decreased sphere formation, although to a lesser extent, was also detected in luminal MCF-7 cells following ATG4*A* inhibition.

### ATG4*A* expression maintains sub-population of cells with cancer stem-cell phenotype

**Figure 5:**
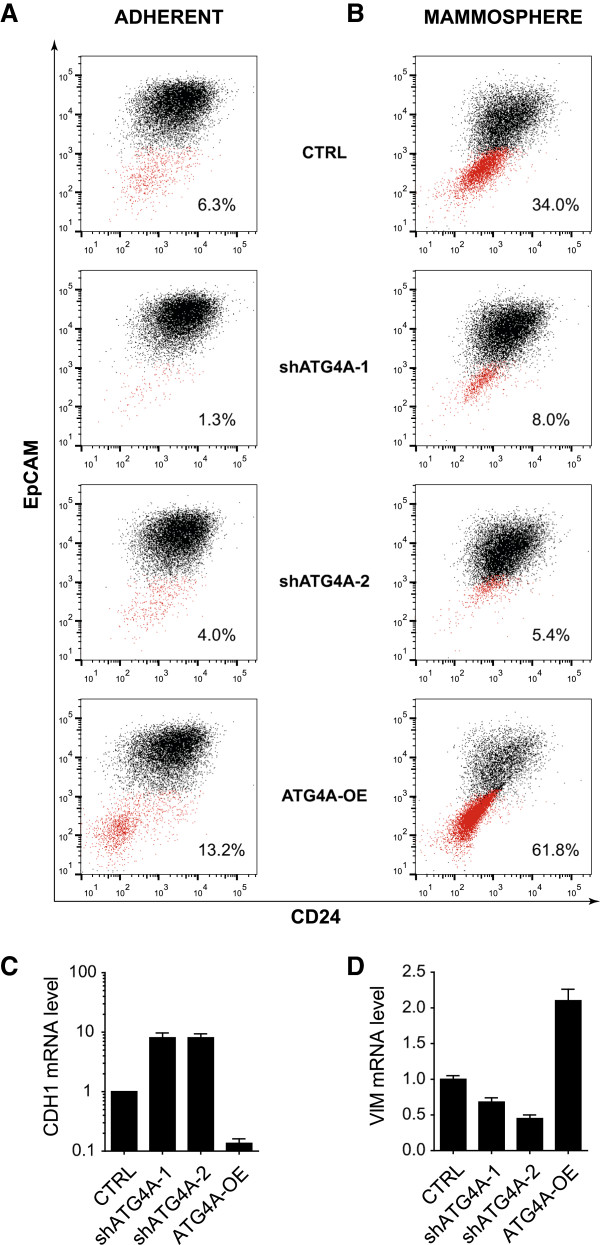
**ATG4A expression regulates maintenance of sub-population with cancer stem-cell (CSC) properties.** Flow cytometry analysis of EpCAM/CD24 expression in SUM-149 cells following ATG4A modulation under **(A)** adherent and **(B)** mammosphere formation conditions. **(C)** CDH1 mRNA level following ATG4A modulation. **(D)** VIM mRNA level following ATG4A modulation. CRTRL, control.

In order to determine the impact of ATG4*A* expression on the sub-population of cells with CSC properties described above, CD24/EpCAM levels were compared between control cells (CTRL) and cells with reduced (shATG4A) or increased ATG4A expression (ATG4A-OE). Under adherent culture conditions, inhibition of ATG4*A* was found to reduce the sub-population whereas its overexpression increased it (Figure 5A). The contribution of ATG4A to the maintenance of this sub-population became even more evident in SUM-149 cells cultured as mammospheres (Figure 5B). Moreover, in mammospheres it was confirmed that ATG4*A* modulation changed mRNA expression levels of CDH1 and VIM. In line with the reduced sub-population following ATG4*A* inhibition, CDH1 levels were found to be increased and VIM levels decreased while the opposite was found for ATG4*A* overexpression (Figure 5C and 5D).

### Modulation of ATG4*A* regulates tumourigenic potential of SUM-149 cells *in vivo*

**Figure 6:**
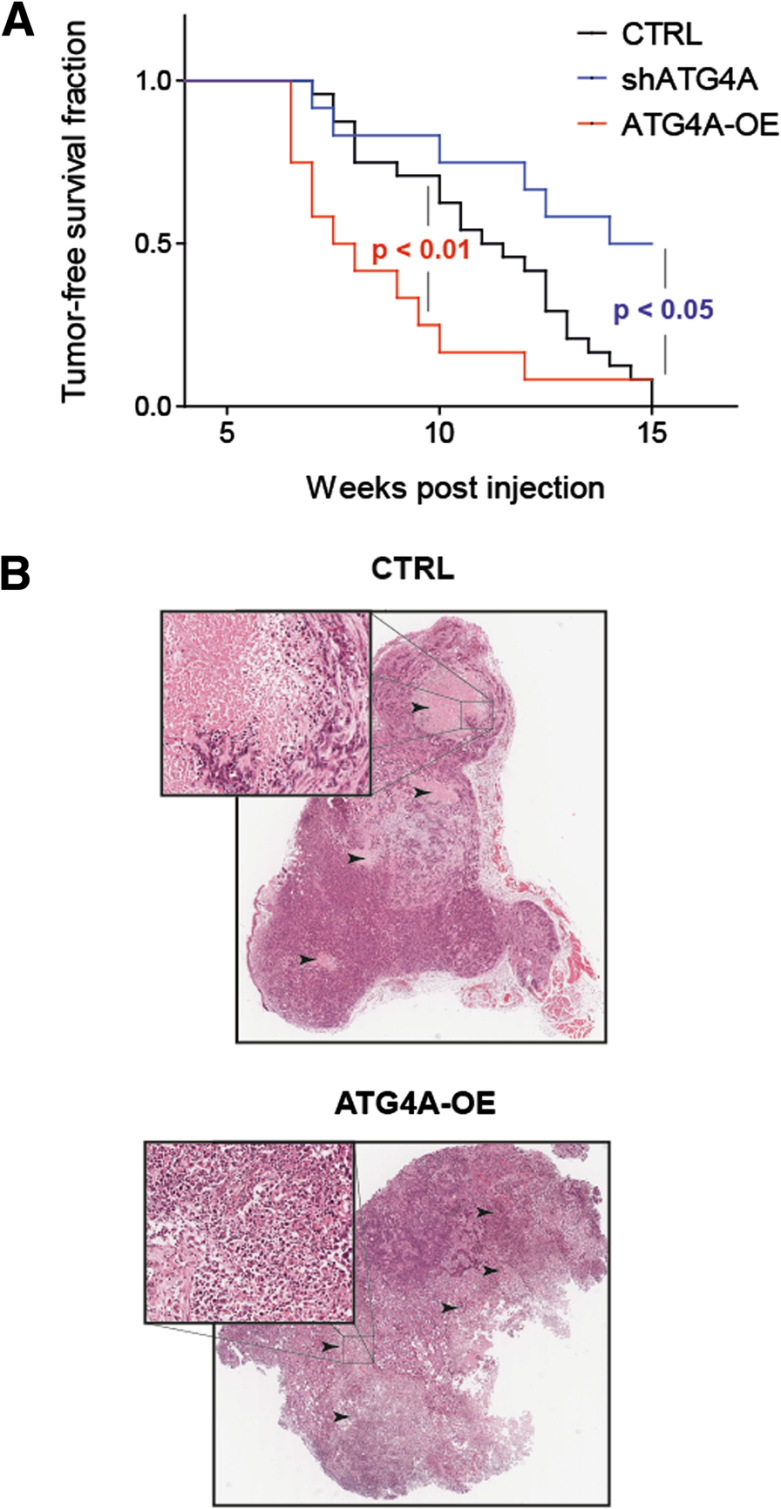
**ATG4A expression regulates tumourigenicity of SUM-149 cells. (A)** Tumour-free survival of NOD SCID gamma mice, following orthotopic injection of 40,000 SUM-149 cells with reduced ATG4A levels (shATG4A), increased ATG4A levels (ATG4A-OE) or control cells (CTRL). The indicated *P*-values were calculated using the Wilcoxon test. **(B)** Representative H&E staining of sections from tumours derived from SUM-149 cells with increased ATG4A expression (ATG4A-OE) or CTRL respectively. Per group, n >10 sections were analysed. Control tumours show few circumscribed necrotic areas with few signs of inflammation, whereas ATG4A-overexpressing tumours exhibit extensive diffuse and strongly inflamed necrotic areas. Arrowheads indicate necrotic areas. Inserts show enlarged necrotic example areas with different degrees of inflammation.

ATG4*A* regulates mammosphere formation in several breast cancer cell lines as well as the maintenance of a sub-population with CSC properties. However, we wondered if it also regulates the tumourigenicity of cancer cells under physiological conditions. To answer this question, SUM-149 cells with inhibited ATG4A expression (shATG4A), cells overexpressing ATG4A (ATG4A-OE) and control cells (CTRL) were injected into the mammary fat pad of NSG mice. Tumour formation was monitored over a period of 15 weeks. As shown in Figure 6A, ATG4A overexpression significantly accelerated tumour formation (*P* <0.01) whereas knockdown caused a reduced tumour burden (*P* <0.05) when compared to control cells. Furthermore, at 15 weeks post injection, 100% of animals from the control group, but only 50% from the ATG4*A* knockdown group, had developed a tumour (Figure 6A). Analysis of H&E-stained tissue sections revealed limited and circumscribed necrosis in control tumours, whereas ATG4A overexpressing tumours displayed extensive and diffuse necrotic areas (Figure 6B). Tumour necrosis is generally associated with rapid tumour growth and was reported as an indicator of poor prognosis in breast cancer [28]. Moreover, the necrotic areas in ATG4A-overexpressing tumours displayed strongly elevated levels of inflammation as judged by the staining of neutrophil granulocytes (Figure 6B, insert). In line with these findings, so-called smouldering inflammation in tumours has recently been described to promote malignant progression [29,30]. Taken together, ATG4A overexpression leads to an increased tumourigenicity of SUM-149 cells in NSG mice and to the development of tumours with a highly aggressive phenotype.

## Discussion

CSCs are rare cells that are suspected to be responsible for tumour recurrence, formation of metastases and chemoresistance [7-9]. The rareness of these cells makes it particularly hard for researchers to study their function. To date, the only functional possibility to enrich breast CSCs with tumour-initiating properties *in vitro* is to culture them as mammospheres [17,18,31]. We found that SUM-149 mammospheres were enriched for cells that expressed a surface marker signature typical for stem-like breast cancer cells (Figure 1E), passed through EMT (Figure 1B and 1F), were chemoresistant (Figure 1C) and more tumourigenic in NSG mice (Figure 1D). These are properties typically attributed to breast CSCs [12]. Although under adherent conditions this sub-population accounted for approximately 6% of the total population (Figure 5A), it became enriched 5-fold in mammospheres (Figure 5B).

Here, we exploited this enrichment to establish a high-throughput pooled RNAi screening system suitable to identify genes that are specifically involved in the maintenance of those rare cells with CSC properties. The system is based on the comparison of two separate RNAi screens performed under (i) adherent and (ii) mammosphere culture conditions (Figure 2A). With the first screen, genes essential for the survival of the total cell population were identified. The second screen identified genes important for sphere formation and hence, served as a surrogate screen to identify genes that are important for the maintenance of cells with CSC properties. Subtractive analysis finally revealed genes that are primarily important for the survival of cells with CSC properties. The identified genes were used for a pathway enrichment analysis, which returned a number of cancer-related pathways and regulatory processes (Table 1B). In total 22 candidate genes were found to be associated with the Jak-STAT signalling pathway (KEGG), making it the most significant pathway identified (*P* = 0.00176). A schematic presentation of some of the identified candidate genes acting in the Jak-STAT pathway is shown in Figure 2C. It is known that cytokine signalling via the IL6 receptor, GP130, JAK3, STAT1 and STAT3, as identified in our screen, is a regulator of breast CSC self renewal and differentiation [32,33]. Further, activated Jak-STAT signalling is essential for the survival of CD44^+^/CD24^-/low^ stem-like breast cancer cells [34] and was shown to play an important role during mammosphere formation [35]. Last, STAT3 was identified by another RNAi screen to be a critical player in mammosphere formation and self-renewal of breast CSCs [36]. Taken together, these findings confirm the utility of the presented screening system to identify processes with specific relevance to the maintenance and expansion of CSCs.

Despite the advantages of a functional enrichment, culturing of cells as mammospheres also has some drawbacks when performing a high-throughput screen. For the analysis of the screen, we pooled all spheres bigger than 40 μm. Therefore, we could not distinguish between sphere size and number of spheres. Large spheres are believed to consist of more differentiated cells or early progenitors than smaller spheres. Another concern might be the formation of spheres because of cell aggregation instead of clonal growth. To overcome this hurdle, we chose an appropriate cell density to avoid any sphere fusion. Moreover, we validated our candidates in semi-solid soft agar colony formation assays that guarantee clonal sphere growth.

Besides Jak-STAT signalling, a number of previously uncharacterized candidate genes were identified in this screen, one of which was the positive regulator of autophagy, ATG4*A*. Autophagy is a lysosome-dependant degradation pathway allowing cells to remove macromolecules and damaged organelles in order to survive stress conditions [37,38]. Interestingly, it was recently published that autophagy promotes the undifferentiated stem-like CD44^+^/CD24^-/low^ phenotype in breast cancer cells [39] and further evidence for the involvement of autophagy in cancer stem-like cell maintenance as well as their differentiation is accumulating rapidly [40-43]. ATG4A is a redox-regulated cysteine protease [44]. ATG4A can cleave ATG8 near its C-terminus allowing the conjugation of ATG8 to phosphatidylethanolamine and subsequently to the membrane of the autophagosome [44]. In a second reaction, ATG4 can delipidate ATG8, releasing it from the autophagosomal membrane [44,45]. This cleavage marks a final step in autophagy and allows the fusion of autophagosome and lysosome [46,47]. The emerging role of autophagy in cancer stem cell maintenance together with the activation of lysosomal gene expression (Additional file 4) and upregulation of ATG4*A* (Figure 4A) in mammospheres strongly suggest an important role for autophagy, or more precisely, ATG4A in breast CSC maintenance. As it was demonstrated here, inhibition of ATG4*A* led to reduction of a sub-population with CSC properties (Figure 5A). Moreover, the enrichment of this sub-population during sphere formation was almost completely prevented by ATG4*A* inhibition (Figure 5B). Furthermore, ATG4A levels influenced size (Figure 3C) and numbers of mammospheres formed from breast cancer cell lines of the luminal and, even stronger, the basal type (Figure 3B and 3E). Last, modulation of ATG4A expression affected the tumourigenicity of SUM-149 cells under physiological conditions in the mammary fat pad of NSG mice (Figure 6A) as well as the composition of resulting tumours (Figure 6B). These results clearly demonstrate that ATG4A is involved in carcinogenesis and the maintenance of cells with a CSC phenotype.

## Conclusion

In order to develop targeted CSC therapies, it is essential to understand the underlying molecular mechanisms of CSC maintenance. To study those mechanisms, we developed a high-throughput negative selection RNAi screening system and provide evidence that it is suitable to identify genes which, like ATG4*A*, are involved in the maintenance of cells with CSC properties. Analysis of additional cell lines using the described approach should greatly accelerate the search for novel molecular targets that could be used to tackle the cancer stem cell.

## Abbreviations

BCA: Bicinchoninic acid; CSC: Cancer stem cell; DMEM: Dulbecco’s modified eagle’s medium; EDTA: Ethylenediaminetetraacetic acid; EMT: Epithelial-mesenchymal transition; FACS: Fluorescence activated cell sorting; GAPDH: Glyceraldehyde-3-phosphate dehydrogenase; H&E: Hematoxylin and eosin; IL: Interleukin; JAK: Janus kinase; KEGG: *Kyoto encyclopedia of genes and genomes*; MOI: Multiplicity of infection; NSG: NOD SCID gamma; PBS: Phosphate-buffered saline; RT-PCR: Reverse transcription polymerase chain reaction; shRNAi: Short hairpin RNA interference; S-P: Sub-population; STAT: Signal transducers and activators of transcription.

## Competing interests

The authors declare that they have no competing interests.

## Authors’ contributions

JW optimised RNAi screening conditions, conducted RNAi screens, performed or participated in all experiments and their analysis, and drafted the manuscript. DLD participated in and performed the validation experiments of ATG4A. JF assisted in the data analysis. KMD performed the xenotransplantation experiments and completed the tissue staining. CF evaluated the immunohistochemical staining. JDH participated in the design of the study and in writing the manuscript. MB designed the study, supervised the experiments and drafted the manuscript. All authors read and approved the final manuscript.

## Supplementary Material

Additional file 1: Table S1 Read-count ratios. Read-count ratios from 27,500 shRNA expression constructs. Click here for file

Additional file 2: Table S2 Average and significance for each gene. Average impact on sphere formation with corresponding *P*-values for all genes. Click here for file

Additional file 3: Table S3 Signalling pathway enrichment RNAi screen. Pathway enrichment analysis of genes identified via mammosphere formation RNAi screen. Click here for file

Additional file 4: Table S4 Signalling pathway enrichment expression profile. Pathway enrichment analysis of genes differentially expressed between adherently cultured cells versus mammospheres. Click here for file
